# Critical dynamics arise during structured information presentation within embodied in vitro neuronal networks

**DOI:** 10.1038/s41467-023-41020-3

**Published:** 2023-08-30

**Authors:** Forough Habibollahi, Brett J. Kagan, Anthony N. Burkitt, Chris French

**Affiliations:** 1 Cortical Labs Pty Ltd, Melbourne, 3056 VIC Australia; 2 Biomedical Engineering Department, University of Melbourne, Parkville, 3010 VIC Australia; 3 Neural Dynamics Laboratory, Department of Medicine, University of Melbourne, Parkville, 3010 VIC Australia; 4 Neurology Department, Royal Melbourne Hospital, Melbourne, Australia

**Keywords:** Learning and memory, Computational neuroscience, Neural stem cells, Dynamical systems, Information technology

## Abstract

Understanding how brains process information is an incredibly difficult task. Amongst the metrics characterising information processing in the brain, observations of dynamic near-critical states have generated significant interest. However, theoretical and experimental limitations associated with human and animal models have precluded a definite answer about when and why neural criticality arises with links from attention, to cognition, and even to consciousness. To explore this topic, we used an in vitro neural network of cortical neurons that was trained to play a simplified game of ‘Pong’ to demonstrate Synthetic Biological Intelligence (SBI). We demonstrate that critical dynamics emerge when neural networks receive task-related structured sensory input, reorganizing the system to a near-critical state. Additionally, better task performance correlated with proximity to critical dynamics. However, criticality alone is insufficient for a neuronal network to demonstrate learning in the absence of additional information regarding the consequences of previous actions. These findings offer compelling support that neural criticality arises as a base feature of incoming structured information processing without the need for higher order cognition.

## Abstract (web summary)

The conditions under which networks of neurons exhibit critical dynamics remains unclear. Here, the authors investigate how simple neural cultures reorganize activity when embodied in a gameplay environment and find that network wide neural criticality arises in nuanced ways.

## Introduction

How do our brains process information? It has been hypothesised for some decades that neural systems operate in or near a “critical state”^1–6^ with well-defined dynamical properties characterised by inter alia, stability of neuronal activity, optimised information storage, and information transmission^4,7^. The presence of “neuronal avalanches” (cascades of propagating activity governed by power laws) as one hallmark of criticality is widely reported in the spontaneous activity of in vivo cortical networks^8–13^. While there is some evidence of neuronal avalanches in vitro in local field potentials (LFPs) of spontaneous activity in slice cultures^5,14^, cultured mouse neurons^15,16^, and neurons differentiated from cultured human stem cells^17^, the characteristics and extent is still unclear. Moreover, the specific role of neural criticality, along with why and when it occurs, remains a matter of significant controversy^18,19^.

Early work identified a link between the balance of excitatory and inhibitory inputs and the critical phase transition^20^. Anticipating this critical transition and the proximity of a network to criticality informs network robustness and can even approximate risk factors of network failures such as epileptic seizures^21^. Moreover, cortical networks express a dynamic equilibrium regime associated with criticality, including: 1) the absence of runaway gains, in which balanced activity is maintained in the neuronal networks such that the neuronal activity does not saturate or become quiescent^3^; 2) a wide coverage in both spatial (mm to cm) and temporal (ms to min, h, etc.) scales during information encoding and transmission^3^; 3) wide dynamical range^22,23^; and 4) maximized information transmission in terms of mutual information^24,25^ and information storage and processing capabilities^26^, such as elevated sensitivity and susceptibility to input. While in the context of population dynamics these criteria have been postulated to be a homeostatic set point for biological neural networks (BNNs), questions about the utility remain^27–30^.

Previous modelling of neuronal avalanches and of cortical slice cultures suggest that information is more optimally transmitted and stored as a result of neuronal networks being tuned near criticality^5,31^. Therefore, criticality has been proposed as a set-point for the self-organization of cortical networks^32^. Nevertheless, some theoretical works have proposed that criticality only benefits the performance in complex cognitive tasks, while resting state conditions are less likely to benefit from these network dynamics^33^. Further support for this view identifies that healthy adults undertaking working memory and cognitive tasks have reported power-law scaling of response time fluctuations^34,35^. Further, some forms of neurological dysfunction have been ascribed to impairment of critical dynamics^36–38^. While indicative, it has also been recognised that power laws are insufficient to infer criticality, since they can emerge from noise^39^. Further findings identifying linkages between criticality and stimulus discrimination, attention, language acquisition, fluid intelligence, and even conscious (awake) behaviour further complicate interpretations^40–46^.

Consequently, there is still a lack of experimental evidence demonstrating whether criticality is a general property of biological neuronal networks, possibly generated by homeostatic mechanisms, or whether it is related to the brain’s response to mere informational load, or a more complex association with cognition. A question that still remains to be answered is whether cortical neuronal networks display a near-critical state during spontaneous activity or whether they only display near-critical states with structured information input - which for in vivo processing would typically occur when undertaking cognitive processing. An additional concern here is that functionally defined neural networks are rarely isolated from the many other connected networks of the intact brain, making it difficult to discern truly local critical functional dynamics as opposed to patterns derived from other regions^47^.

To determine how criticality may arise without these overlapping compensatory mechanisms requires simplified models that are able to be presented and respond to structured information. To address this requirement and better evaluate these questions on the role of neural criticality, data was analysed from an in vitro neural network of cortical neurons which was trained to play the game ‘Pong’. We utilized *DishBrain*, a novel system shown to display goal-directed activity changes by harnessing the inherent adaptive computation of neurons to show what has been termed Synthetic Biological Intelligence (SBI)^48^. As such, this work also serves as a useful demonstration of the utility of these closed-loop SBI systems over spontaneous activity alone. We hypothesise that near-critical network behavior emerges when neural networks receive structured sensory input and that this system would develop a network structure closer to critical states with successful task acquisition.

## Results

Cortical cells, either differentiated from human induced pluripotent stem cells (hiPSC) or derived from E15 mouse embryos, were subjected to the *Gameplay* and *Rest* conditions in the *DishBrain* system as previously described in ref. ^48^. Hit-to-miss ratio and distance from the critical state were compared in different experimental conditions - see Supplementary Information section 1.1–1.4 and Supplementary Fig. S1. The measurements were carried out in both the conditions of (i) *Gameplay*, where cells adjusted paddle position through activity changes and received information about the position of the ball and the closed-loop response to their control of it, and (ii) *Rest*, where neuronal activity adjusted the paddle position, but received no input, in order to give a matched control. For more details, see Supplementary Information section 1.4–1.5 and Supplementary Fig. S3.

**Fig. 1:**
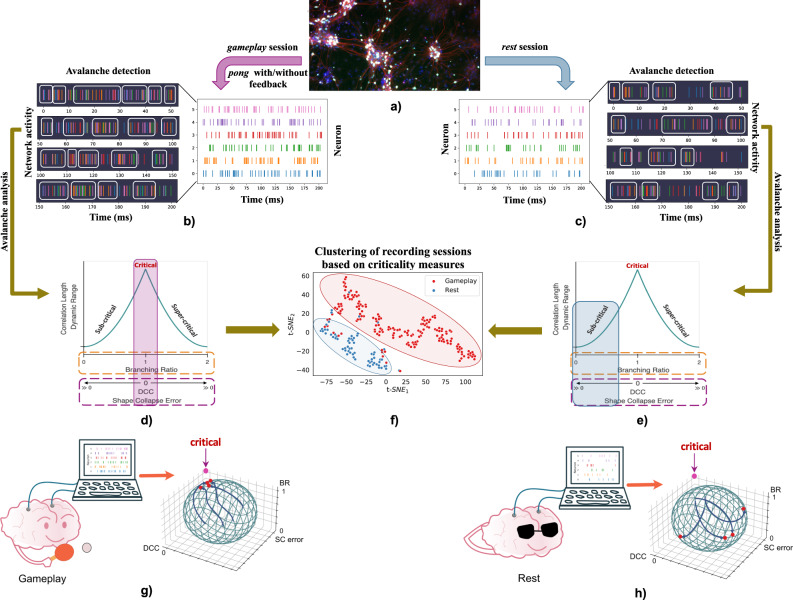
Schematic overview of study. **a)** Showing cortical cells harvested from embryonic rodents. **b)** & **c)** The recorded population activity from these cortical cells is then binned to 50 ms bins during both *Gameplay* and *Rest* sessions. The neuronal avalanches are cascades of network activity that surpass a certain activity threshold for a certain duration of time, which are then extracted by bin. **d)** & **e)** Avalanches are utilized to examine the criticality metrics in the neuronal network’s activity patterns to identify the working regime of each recording in terms of being sub-, super-, or near-critical. **f)** The same measures of criticality are used to cluster the recordings between two groups of *Gameplay* and *Rest*. **g**) & **h)** Illustration of the experimental pipeline in which cultured cortical networks are recorded during *Gameplay* and *Rest* states. The recorded neuronal activities are then employed to extract the 3 metrics of criticality (namely Branching Ratio (BR), Deviation from Criticality Coefficient (DCC), and Shape Collapse error (SC error)) which are found to move towards the critical point during *Gameplay* **g**) and move further from that point during *Rest* **h**).

Neuronal avalanches were identified in network recordings. The scale-free dynamics of detected neuronal avalanches, as well as the Deviation from Criticality Coefficient (DCC), Branching Ratio (BR), and Shape Collapse error (SC error) were evaluated to identify whether the recordings were tuned near criticality. Figure 1a–f provide a visual overview of the framework utilized in this study to investigate how far the dynamics of in vitro networks of cortical neurons are from criticality and whether this distance can accurately distinguish between task-present and task-absent states being processed by the neurons. Table 1 summarises these metrics of criticality and their formulations (see Section 'Data analysis' and Supplementary Figures S6, S7 and refs. ^3, 49, 50^). At criticality, BR of the network is tuned near 1.0 while DCC and SC error diminish to 0.

**Table 1:** Criticality parameters and metrics with details of their formulation

Notation	Definition	Formulation
*α*	Calculated exponent for the truncated power law distribution fitted on avalanche duration, *D* (time).	$f(D)=\frac{{D}^{-\alpha }}{\mathop{\sum }\nolimits_{{D}_{\min }}^{{D}_{\max }}{D}^{-\alpha }}$, where maximum likelihood estimation was used to fit a truncated power law to the avalanche duration distribution (*f*(*D*)).
*τ*	Calculated exponent for the truncated power law distribution fitted on avalanche size, *S* (number of spikes).	$f(S)=\frac{{S}^{-\tau }}{\mathop{\sum }\nolimits_{{S}_{\min }}^{{S}_{\max }}{S}^{-\tau }}$, where maximum likelihood estimation was used to fit a truncated power law to the avalanche size distribution (*f*(*S*)).
*β*_pred_	The third hidden power law exponent in critical systems which represents the relationship between size and duration exponents.	${\beta }_{{{{{{{{\rm{pred}}}}}}}}}=\frac{(\alpha -1)}{(\tau -1)}$.
DCC	Deviation from criticality coefficient.	DCC = ∣*β*_pred_ − *β*_fit_∣, where $\langle S\rangle \propto {D}^{{\beta }_{{{{{{{{\rm{fit}}}}}}}}}}$.
BR	Branching ratio is the ratio of the number of neurons spiking at time step *t* + 1 to the number of active neurons at time step *t*.	〈*N*(*t* + 1)∣*N*(*t*)〉 = BR ⋅ *N*(*t*) + *h*, where *N*(*t*) is the number of active neurons at time *t* and *h* is the external drive.
SC error	Avalanche profiles of all sizes are copies of each other as they unfold from different scales, and they all collapse to the same universal shape. A collection of scaling functions (*F*(. )) are extracted for various *D* durations. The error for this process is described as: $\frac{{{{{{{{\rm{var}}}}}}}}(F)}{{(\max (F)-\min (F))}^{2}}$.	$s(t,D)\propto {D}^{\gamma }F\left(\frac{t}{D}\right)$, where $\langle S\rangle (D)=\int\nolimits_{0}^{D}s(t,D)dt,F\left(\frac{t}{D}\right)$ is a universal function for all avalanches, *γ* = *β* − 1, and SC error is ∣*β* − *β*_pred_∣ when $\frac{{{{{{{{\rm{var}}}}}}}}(F)}{{(\max (F)-\min (F))}^{2}}$ is minimised.

### Cultured cortical networks show markers of criticality when engaged in a task but not when resting

Data from 14 different cultures integrated on HD-MEAs during 308 experimental (192 *Gameplay*; 116 *Rest*) sessions were recorded and discretized into 50 ms bins. For full details about the number of cultures and number of experiments performed on each culture, please see Supplementary Information section 1.6 and Supplementary Fig. S5. The sum of activities from all the recording channels in each time bin denotes the network activity. The network state was then evaluated using each of the described measures of criticality.

**Fig. 2:**
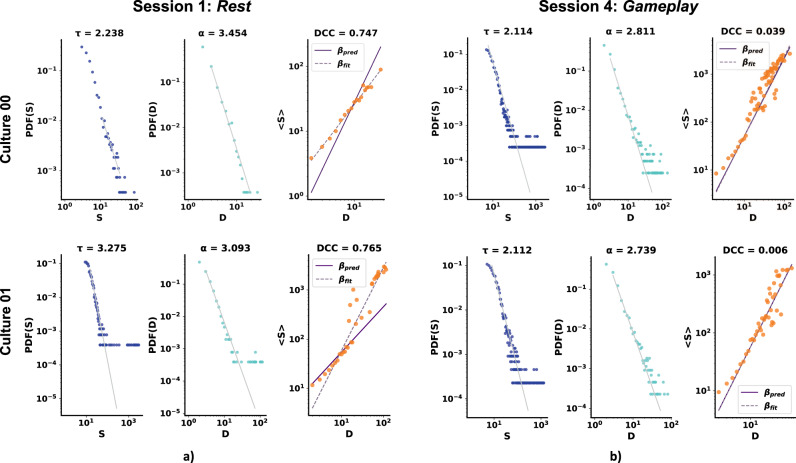
Culture dynamics vary drastically when receiving structured information through gameplay related stimulation. Avalanche size and duration PDF plots and the calculated DCC values for 2 representative sample cortical cultures at **a**) *Rest* (i.e. Session 1) and **b) ***Gameplay* (i.e. Session 4) of the same experiment and the corresponding *α* and *τ* exponents. DCC is given by ∥*β*_pred_ − *β*_fit_∥; for details, see Table 1 and section Exponent relation and Deviation from Critically coefficient (DCC).

Figure 2 illustrates the fitted PDF functions to avalanche size and duration and the associated pair of exponents (*τ* and *α*); the exponent is the slope of the line in a log-log plot. The associated DCCs extracted from the network are also represented. Data from two sample cultures are displayed to illustrate the comparison between the network’s dynamical state during *Rest* and *Gameplay*. Fitted power law distributions, DCC values, and the span of distributions in both size and duration domains are visualized in a *Rest* session (e.g., Session 1) against a *Gameplay* session (e.g. Session 4.)

Additionally, BR and SC error were also extracted for all cultures in recording sessions 0 to 4.

**Fig. 3:**
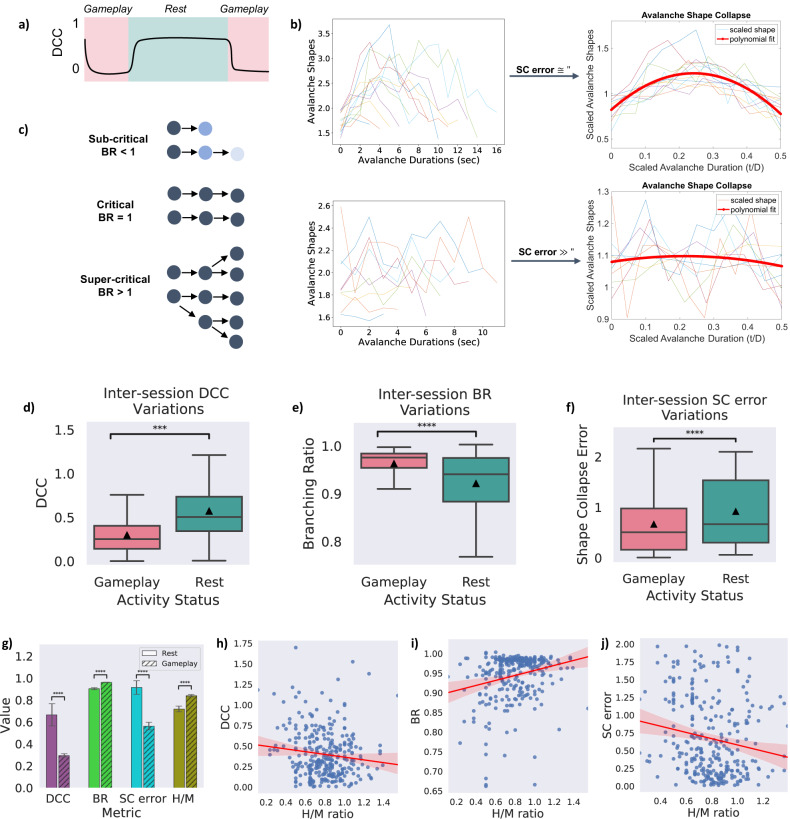
Comparison between critical and non-critical dynamics. **a)** Illustration of the course of the expected change in the DCC measure when transitioning between near-critical and non-critical regimes. **b)** Comparison of the shape collapse error while scaling avalanche shapes in 2 sample recording sessions. Scaled avalanches across a range of durations show little error around the polynomial fit in the upper row (indicative of a near-critical regime) while this error increases significantly in the data represented at the bottom row (indicative of a non-critical regime). **c)** Effect of branching ratio (BR) on activity propagation through a network over time. In critical regimes, BR = 1.0 and, on average, activity neither saturates nor decays across time. **d**–**f)** DCC, BR, and SC error extracted for all the recordings and compared between *Gameplay* and *Rest*. The illustrated trend in all measures supports the conclusion of the system tuning near criticality during *Gameplay*. The *Gameplay* recordings display DCC and SC error values closer to 0 and branching ratios closer to 1; features which are missing in the *Rest* recordings. Box plots show interquartile range, with bars demonstrating 1.5X interquartile range, the line marks the median and the black triangle marks the mean. Error bands, 1 SE. *** indicates *p* < 5 × 10^−4^ and **** indicates *p* < 5 × 10^−5^. **g)** Summary of the key characteristics of a critical system compared between all *Rest* and *Gameplay* sessions as well as the corresponding performance level in terms of the observed H/M ratio. Error bars, SEM. **** indicates *p* < 5 × 10^−5^. The sample sizes of the box and bar plots are equal to the number of independent *Gameplay* recordings (*n* = 192) and *Rest* recordings (*n* = 116). Alexander-Govern approximation test with *p* = 7.836*e* − 06, *p* = 5.667*e* − 13, *p* = 2.460*e* − 07, and *p* = 3.356*e* − 06 for DCC, BR, SC error, and H/M ratio in *Gameplay* vs *Rest*. **h)** A weakly significant negative correlation was found between DCC and the neuronal culture performance in terms of H/M ratio (*r* = − 0.13, *p* < 0.05, Pearson Correlation test). **i)** A strongly significant positive association was observed between BR and H/M ratio (*r* = 0.24, *p* < 0.00005, Pearson Correlation test). **j)** A strongly significant negative correlation was found between SC error and H/M ratio (*r* = − 0.17, *p* < 0.005, Pearson Correlation test). Shades represent the 95% confidence intervals. Source data are provided as a Source Data file.

Figure 3a–c illustrate a general comparison between the critical and non-critical dynamics in terms of each of the introduced criticality metrics, DCC (3a), BR (3b), and SC error (3c). An Alexander-Govern approximation test was run to investigate the significance of the differences between the two groups for each extracted metric. Figure 3d–f illustrate the distribution of the criticality metrics in different recording sessions of the experiments. Comparison of the *Rest* (colored in teal) and *Gameplay* (colored in pink) sessions indicates the shift of cultured cortical network dynamics towards criticality during the task-present sessions. The *Gameplay* “Hit to Miss Ratio” (H/M ratio) - the number of accurate “hits” to the number of “missed” balls - was also found to be significantly higher than during *Rest*. A summary of the statistical comparisons including the comparison of H/M ratio is given in Fig. 3g.

These results indicate the shift towards self-organized criticality of the neural cultures in these experiments when exposed to external structured information such as the game environment of ‘Pong’. In contrast, cultured cortical networks deviated from the critical state during *Rest* sessions when the paddle was solely affected by the neurons’ spontaneous activities. When the cells were not presented any external information about the status of the ball or the game (such as in *Rest* conditions), the network parameters indicated a sub-critical system. These results suggest that during task-present conditions (here accompanied by learning, which is reflected in the improved H/M ratio of experimental cultures), the cultured cortical network tunes itself near criticality.

Notably, deviation from criticality was also measured in time-shuffled data acquired from the *Gameplay* sessions. These data preserved the spatial correlations but randomized the temporal structure and obtained a significantly higher DCC value compared to the original data, indicating a larger deviation from criticality compared to the original data (DCC for time shuffled and original recordings: 0.627 ± 0.087 and 0.296 ± 0.015 respectively, *p* < 0.0005, Alexander-Govern approximation test). Specifically, we sought further control data by analysing time-shuffled data to detect the sensitivity to the ensemble activity’s temporal structure. This control is important since the comparison can then eliminate the potential role of temporal random effects in detecting the critical dynamics.

Furthermore, to determine whether the identified criticality metrics correlated with game performance, exploratory uncorrected Pearson’s correlations were computed for criticality metrics and H/M ratio for all recording sessions (see Fig. 3h–j). While a significant negative correlation was found between DCC and H/M ratio (*r* = − 0.13, *p* < 0.05, Pearson Correlation test) as well as SC error and H/M ratio (*r* = − 0.17, *p* < 0.005, Pearson Correlation test), a strong positive association was observed between BR and culture performance represented by H/M ratio (*r* = 0.24, *p* < 0.00005, Pearson Correlation test). This indicates that network dynamics closer to criticality may be related to better performance.

### Culture *Gameplay* vs *Rest* status is predicted by criticality metrics and H/M ratio

**Fig. 4:**
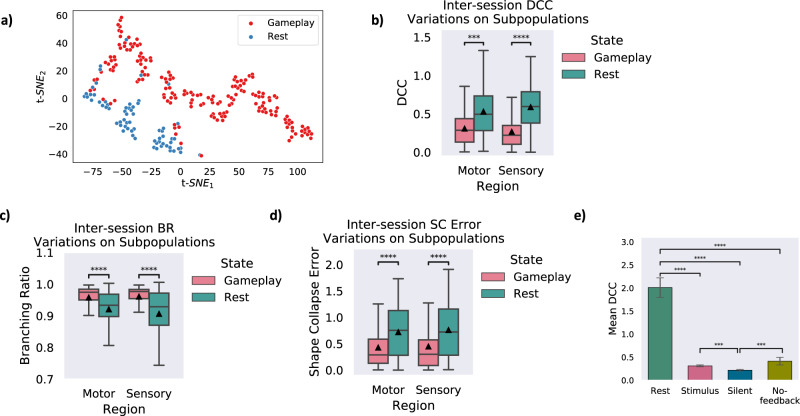
Critical dynamics are observed in subpopulations of neurons during *Gameplay* but not *Rest* and in different feedback conditions. **a)** Visualization of the extracted representation for each data point using the t-SNE algorithm in a 2-dimensional space (i.e., dimensions t-*SNE*_1_ and t-*SNE*_2_). The two *Rest* and *Gameplay* classes are illustrated with different colors. **b)** DCC, **c**) BR, and **d**) SC error variations between *Rest* and *Gameplay* sessions in separate motor and sensory regions of the cultures. The illustrated trend in all three measures on the subpopulations is in line with the previous conclusion about the entire population. A similar pattern in these results also states that during *Gameplay* the neuronal ensembles move near criticality while in *Rest*, they are further from a critical state. ***p < 10^−3^, ****p < 10^−5^. Box plots show interquartile range, with bars demonstrating 1.5X interquartile range, the line marks the median and the black triangle marks the mean. Error bands, 1 SE. The sample sizes of the box and bar plots are equal to the number of independent *Gameplay* recordings (*n* = 192) and *Rest* recordings (*n* = 116). Alexander-Govern approximation test with *p* = 8.172*e* − 4,  *p* = 8.839*e* − 6, and *p* = 7.139*e* − 6 for DCC, BR, and SC error in the motor region and *p* = 5.627*e* − 12, *p* = 4.637*e* − 7, and *p* = 1.442*e* − 6 for DCC, BR, and SC error in the sensory region in *Gameplay* vs *Rest*. **e)** Comparing the average DCC measure calculated in different feedback conditions with the *Rest* sessions. ***p < 5 × 10^−3^, ****p < 10^−10^. Error bars, SEM. The sample sizes of the bar plots are the number of independent recordings during *Rest* or different feedback conditions, that is *n* = [209, 113, 119, 95] for *Rest*, *Stimulus*, *Silent*, and *No-feedback*. Source data are provided as a Source Data file.

Binary classification of the data was performed to predict group membership of each recording session and assign it to either the *Rest* or *Gameplay* classes. Three different classification algorithms were utilized: Logistic Regression, Support Vector Machines (SVM), and Random Forests. Table 2 represents the mean prediction accuracy for various classification methods as well as different approaches in assigning feature vectors to the data points. 4-Metrics refers to the case where a 4 dimensional vector of all the 4 metrics represented in Fig. 3g were used to represent each data point. 3-Criticality metrics indicates a case where only 3 criticality metrics are used to form the feature vectors. The conditions where each metric is separately used to represent the data points is also included. The results demonstrate that the highest accuracy of prediction can be achieved using all 4 criticality metrics accompanied by the culture’s H/M ratio. Nevertheless, it was also found that merely employing the criticality measures is sufficient for an accurate prediction (up to 92.41%) of the culture’s status in terms of it being task-present or task-absent (i.e., the default resting state). These findings suggest that knowledge about a neuronal network’s distance from criticality may be adequate for distinguishing between task-present and task-absent states and whether the input information is being optimally processed. Data representations were visualized using the obtained feature vectors. Since the 4-Metrics representation proved to be the most effective representation given the results in Table 2, we considered this case for the visualization task. A standard t-SNE algorithm^51^ visualized the data representations as per Fig. 4a. A 2-dimensional visualization of the sessions is obtained with each recording session represented as a colored dot. The pairwise dissimilarities between each data point (i.e. each recording session) and their corresponding projections in the resulting 2-dimensional mapping were then calculated. The Kullback-Leibler divergence as a measure of this dissimilarity between distributions was 0.373, which indicates an accurate network representation.

**Table 2:** Comparison of the mean prediction accuracy for different binary classifiers on all the recorded sessions

Classifiers	Feature Vectors					
	4-Metrics	3-Criticality metrics	DCC	BR	SC error	H/M ratio
Logistic Regression	0.8734	0.7215	0.7215	0.7215	0.7215	0.8861
SVM	0.962	0.7932	0.7296	0.7342	0.7253	0.9241
Random Forest	0.9821	0.9241	0.8043	0.8261	0.8043	0.9565

### Motor and sensory subpopulations inherit the criticality characteristics of the entire neuronal ensemble

In the *DishBrain* system configuration, a specific frequency and voltage are applied to key electrodes in the predefined sensory areas, as described in ref. ^48^. Then, different predefined motor region configurations are examined to select the configuration that maximises performance. The paddle moves in a corresponding direction based on the region with the higher activity (For more details, please see Supplementary Fig. S2).

We assessed the activity dynamics of the overlaid neuronal subpopulations on each of the predefined region of electrodes. Consequently, the introduced criticality metrics were measured from the recorded activities of each of these subpopulations separately. Figure 4b–d demonstrates that these subpopulations also exhibit similar features of a near-critical system when exposed to the *Gameplay* setting. Yet both motor and sensory neuronal populations were identified to be statistically significantly closer to criticality compared to *Rest* session recordings from the same neuronal subpopulations (*p* < 10^−3^ for DCC in the Motor subpopulation and *p* < 10^−5^ for all other comparisons, Alexander-Govern approximation test). Supplementary Table S1 presents full details on all multivariate statistical tests performed in relation to these figures.

### Feedback is required for improved game performance in a critical system

Biological neuronal networks typically require feedback for learning to occur - i.e., a closed-loop between action and consequence. In a closed-loop system, feedback is provided on the causal effects of the neuronal culture’s behavior^48^. Three different feedback conditions were employed in this study. Condition 1 (*Stimulus*), is where predictable and unpredictable stimuli are administered when the cultures behaved desirably or not, respectively (results reported previously). Condition 2 (*Silent*), involves the above stimulus feedback being replaced with a matching time period where all stimulation was withheld. Condition 3 (*No-feedback*), involves a more drastic change to the gameplay environment, where the ability for the ball to be missed was removed, so when the paddle failed to intercept the ball, the ball bounced instead of triggering a reset, and the game continued uninterrupted^48^ (see Supplementary Information section 1.4 and Supplementary Fig. S4). Performance of the cultures in terms of their H/M ratio, as well as their criticality characteristics, were measured under all three feedback conditions and then compared to the *Rest* sessions. Table 3 represents the results (Mean ± SE) for 14, 15, and 12 different cultures under *Stimulus*, *Silent*, and *No-feedback* conditions respectively. These additional experiments were done beyond the recordings used in the previous analysis (For full details about the number of cultures and number of experiments performed on each culture in each feedback type, please see Supplementary Information section 1.6 and Supplementary Fig. S5). Overall 113, 119, and 95 sessions were recorded under the *Stimulus*, *Silent*, and *No-feedback* conditions respectively, and were compared to 209 *Rest* sessions obtained from the total of 41 cultures under experiment. The reported *p*−values represent the significance of the difference between the obtained measures in each feedback condition and the *Rest* cultures. It is very interesting to observe that the *Silent* condition shows significant performance in the game (H/M ratio) as well as showing dynamical features that are indicative of a near-critical system. The deviation from criticality (DCC) is significantly lower in the *Silent* condition compared to *Stimulus* or *No-feedback* (*p* < 0.005, Alexander-Govern approximation test) conditions (see Fig. 4e. This difference was not significant when comparing *Stimulus* and *No-feedback* conditions. While comparing the gameplay characteristics of the cultures (H/M ratio), the *Stimulus* and *Silent* conditions both significantly outperform the *No-feedback* conditions (*p* < 0.0005 and *p* < 0.005, Alexander-Govern approximation test). While the *No-feedback* system also represents features characterizing near-critical dynamics (although to a lesser extent compared to the other two closed-loop systems), the game performance significantly deteriorates in this case (no significant outperformance compared to the *Rest* state, *p* = 0.085, Alexander-Govern approximation test). This demonstrates that fine-tuning near criticality may be necessary for optimal information processing when facing an increased load. Nonetheless, criticality may not be sufficient for a neuronal network to achieve its learning and memory goals in the absence of additional information regarding the consequences of previous actions, i.e., feedback. All details for *post-hoc* follow-up tests in relation to this figure are presented in Supplementary Table S2.

**Table 3:** Comparison of the 4 extracted criticality measures as well as the H/M ratio for the *Rest* and *Gameplay* groups

	Rest	Stimulus	Silent	No-feedback
DCC	2.010 ± 0.212	0.311 ± 0.020 (*p* < 10^−13^)	0.217 ± 0.017 (*p* < 10^−14^)	0.414 ± 0.082 (*p* < 10^−11^)
BR	0.914 ± 0.008	0.956 ± 0.003 (*p* < 10^−4^)	0.970 ± 0.002 (*p* < 10^−4^)	0.960 ± 0.004 (*p* < 10^−4^)
SC error	0.970 ± 0.033	0.591 ± 0.036 (*p* < 10^−10^)	0.600 ± 0.039 (*p* < 10^−10^)	0.621 ± 0.040 (*p* < 10^−7^)
H/M ratio	0.749 ± 0.018	0.898 ± 0.021 (*p* < 10^−4^)	0.868 ± 0.024 (*p* < 10^−4^)	0.787 ± 0.012 (*p* = 0.085)

The *p-*value of an Alexander-Govern approximation test is reported for each measure in comparison of each feedback condition with *Rest* sessions. Source data are provided as a Source Data file.

### Critical dynamics show nuanced differences based on bursting patterns of activity

Prior studies have demonstrated that dissociated cortical cell cultures display a diverse range of activity patterns^52^. The bursting patterns observed in these cultures exhibit developmental changes and significant variability across different cultures. These findings highlight the value of utilizing multiple preparations in any investigation involving neuronal cultures. We utilized the burst (or avalanche) classification methods and metrics introduced by^52^ to extract quantitative details from the detected avalanches during *Rest* recordings. Our aim was to distinguish between cultures based on their bursting patterns and investigate whether any of the criticality metrics examined in our research exhibited significant differences between the different classes of cultures. The following criteria were extracted from the spontaneous activity of the cultures during the *Rest* recordings: 1) Size distribution of avalanches, 2) Burst (avalanche) rates, and 3) Superbursts. These measures were employed as classifiers to identify the bursting pattern of each culture. For definitions of these criteria and their quantification, see Section 'Burst pattern analysis' and ref. ^52^.

**Fig. 5:**
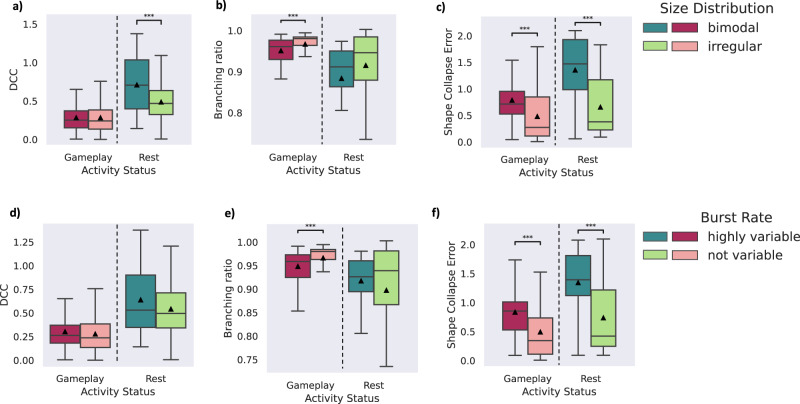
Comparison of criticality metrics between different bursting patterns for either *Gameplay* or *Rest*. **a**–**c)** DCC, BR, and SC error of all *Rest* and *Gameplay* sessions for different size distributions of avalanches observed during *Rest* recordings of each culture. **d**–**f)** DCC, BR, and SC error of all *Rest* and *Gameplay* sessions for different types of burst rates observed during *Rest* recordings of each culture. Alexander-Govern approximation test was utilized. ***p < 5 × 10^−3^ with **a**) *p* = 8.335*e* − 4 during *Rest*, **b**) *p* = 4.705*e* − 3 during *Gameplay*, **c**) *p* = 1.064*e* − 4, and *p* = 6.423*e* − 8 during *Gameplay* and *Rest* respectively, **e**) *p* = 4.815*e* − 3 during *Gameplay*, and **f**) *p* = 9.514*e* − 5, and *p* = 1.068*e* − 5 during *Gameplay* and *Rest* respectively. Box plots show interquartile range, with bars demonstrating 1.5X interquartile range, the line marks the median and the black triangle marks the mean. Error bands, 1 SE. The sample sizes of the box plots are equal to the number of independent *Gameplay* recordings (*n* = 192) and *Rest* recordings (*n* = 116). Source data are provided as a Source Data file.

Based on our measurements, we observed that none of the recordings in our dataset exhibited superbursts during the *Rest* state. However, there were notable differences in terms of the size distribution and burst rates among the recordings. Specifically, two types of size distributions, ‘bimodal’ and ‘irregular’, were identified across various recordings (no ‘fixed’ distributions were found). Additionally, the burst rates were classified into two groups, ‘highly variable’ and ‘not variable’. Figure 5 illustrates the DCC, BR, and SC error extracted for all the recordings during *Gameplay* and *Rest*, and compares them between different classes of size distribution or burst rates. Supplementary Table S1 presents full details on all multivariate statistical tests performed in relation to this figure.

During the *Gameplay* sessions, several metrics did not exhibit a statistically significant difference between the classes. However, the BR and SC error metrics showed indications of being in a closer proximity to a critical state, as evidenced by higher BRs and lower SC errors, when avalanches had an ‘irregular’ size distribution and ‘not variable’ burst rates. In a near-critical system, we anticipate observing avalanches of various sizes that span multiple orders of magnitude. The presence of an ‘irregular’ size distribution during *Rest* is suggestive of a system in which bursts of varying sizes occur, and is consistent with the hallmarks of a system that can achieve near critical dynamics. Therefore, this observation could be expected. Moreover, the findings suggest that the ‘not variable’ burst rates in the spontaneous activity of the cultures were also indicators of cultures that achieved more proximate dynamics to criticality.

Based on analysis of the *Rest* state recordings, the ‘irregular’ size distribution exhibited indications of being in closer proximity to criticality, as evidenced by lower DCC and SC error. This data provides a nuanced perspective for how spontaneous electrophysiological characteristics may influence the expression of criticality dynamics in vitro. The difference in criticality at *Rest* for cultures with ‘irregular’ and ‘not variable’ firing patterns is suggestive that these cultures may have more complex interconnectedness that could facilitate critical dynamics even at *Rest*. Yet, when embodied in a structured information landscape through gameplay, the differences between these cultures are greatly ameliorated, potentially because more complex informational flows are available to the culture through the simulated environment. Nevertheless, these results should be considered as preliminary evidence at best, as the lack of concordance between all metrics for criticality prevents any robust conclusions.

## Discussion

When considering how neural systems process information, near-critical dynamics in the brain remain a fascinating phenomenon. The primary hypothesis that near-critical network behaviour emerges when neural networks receive structured sensory input was strongly supported. Criticality was readily observable for in vitro neuronal cultures when embodied in a virtual environment^48^ through structured stimulation. As evidenced through multiple features expected of a near-critical system, we found that cultured networks of cortical neurons self-organized to display these key markers when receiving structured information, but not when unstimulated. Through this it was robustly observed that in vitro cortical neurons exhibited markers of criticality when actively engaged in a task and receiving feedback contingent on neuronal activity modulating the simulated world.

Comparing the data from this study with previous literature investigating neural criticality in vitro, some key observations can be made. Previously most studies finding evidence of criticality in vitro conclude that criticality would arise in some cultures after maturation^5,15–17^. Consistent with this, we did discover closer to critical dynamics on some metrics in cultures with certain bursting patterns - specifically those that exhibited more variable and irregular activity. Whether this is occurring in cultures that showcase more complex networks is unclear and beyond the scope of this single study, yet forms an important direction for future research. Nevertheless, here we found that in vitro neuronal networks show particularly robust markers of criticality only when presented with structured information through electrical stimulation. In contrast, while in the default resting state of in vitro neuronal networks, i.e., not embodied within a game environment, despite spontaneous activity exhibiting neuronal avalanches, cultures no longer display dynamics that were as close to criticality across all metrics. This finding that even though evidence of avalanches were identified within spontaneous activity, they did not show consistency across more robust measures of criticality, may explain the difference between this work and the previously described in vitro measures of criticality that focused predominately on power laws. While relying on identifying power-law scaling in temporal and partial domains is common historically^34,35^, power laws have more recently been shown to also have the potential to emerge from noise^39^. Better practice is to have power laws accompanied by independent stochastic surrogates, such as disconnected nodes in a complex system^39^. Here we co-analysed the described three established markers of criticality on spiking data generated in this system^39,49^. We observed an exceptionally high degree of qualitative concordance between these different measures, adding confidence to the internal validity of the results. Likewise, we find the extent of this difference based on markers of criticality alone was stark enough to predict whether a given culture was actively engaged in gameplay or resting with a 92.41% accuracy. When performance data was included this accuracy increased to 98.21%, further supporting the dramatic difference between resting and active cultures. The additional finding that these markers of criticality were persistent across sub-populations defined by their external relationship to the game-world for the neuronal cultures, suggests a network-wide coordination of activity. This does not mean that no critical dynamics were present in previous work^5,15–17^, rather it may suggest that there are differences in the extent of critical dynamics under different conditions, where criticality is best conceptualised as a spectrum. Therefore, it can be coherently accepted that as the neural cell cultures matured, they possibly underwent a transition towards critical dynamics without external stimulation. Furthermore, by placing the cultures in a closed-loop structured information environment, they may have moved even closer to criticality. Given the importance of the excitatory-inhibitory balance in maintaining critical dynamics^20,53^ coupled with tendency for inhibitory pathways to become more prominent over time^54^, such a finding would indeed be expected and forms an interesting direction for future research.

Furthermore, the finding that significantly closer to critical dynamics are observed in vitro when cortical networks are integrated with in silico computing via HD-MEA to experimentally explore the notion of criticality under task-present compared to task-absent states can also be applied to a in vivo context. It has been proposed that certain features of learning, including information capacity and transmission, are optimized at criticality^55^. Indeed, many studies have identified in vivo that cortical networks typically function near a critical point^4,5^, the extent of which shows key correlations with performance^3^. Taken in concert with past research identifying power-law-like behaviour in brain activity of humans undergoing cognitive tasks, this is indicative of a network-wide fundamental computation underlying information processing which may be ongoing in these cultures only when actively engaged in a task or otherwise presented structured information^34,35^. For example, the importance of critical state dynamics in language acquisition has been highlighted^42,43^. Moreover, resting-state fMRI data of neurotypical adults with varying IQs has found a connection between high fluid intelligence and close proximity to a critical state in a spin-glass model^40^. In addition, conscious states of mind have been linked to near-critical slow cortical electrodynamics, suggesting that the disruptions in information processing during unconscious states are due to the transition of low-frequency cortical electric oscillations away from the critical point^41^. In contrast, several studies demonstrate more ambiguous results around the relationship between electrical brain response to increased cognitive load^56–59^. Moreover, while numerous studies have observed critical dynamics from spontaneous activity in vivo^8–13^, the neural regions measured are not isolated from external input, including input from other unmeasured neural regions, even when anesthetized. This makes it infeasible to determine whether in vivo neural systems are spontaneously tuned towards critical dynamics, or if this arises through a dynamic interplay between systems where critical points can generalize to critical regions^60^. Moreover, in the majority of these previous studies, the observation of power laws was regarded as the primary indicator of criticality. As noted above, the criticisms of this approach^49,61,62^ makes it infeasible to robustly establish that these in vivo neural systems are certainly operating at criticality, or to what extent criticality is displayed relative to other states if so.

Our results can also be compared to the increasing evidence linking near-critical dynamics in the brain with cognitive-like behaviour^63–65^. The secondary part of our hypothesis was that the neural cell activity would develop a network structure closer to a critical state with successful task acquisition. Although there was some support for this hypothesis, the data suggests a more fundamental role of criticality, where criticality may be considered a necessary but not sufficient conditions for dynamic cognitive-like behaviours - such as successful task acquisition to arise. While the results did find consistent and significant positive correlations between performance and critical dynamics, it was also found that under different conditions that did not result in learning (i.e., the *No-feedback* condition), neural activity was still closer to criticality than at rest. Yet, rather than challenging these previous studies, we believe this data offers a pathway to unify results under a broader perspective. Specifically, that criticality is not tied to general processing, learning, or cognition, but is rather optimised for specific tasks or types of information processing. Previously, criticality was found to be linked to stimulus discrimination, yet decreased stimulus detection^44^. In our data this would be supported by the observed variation in mean DCC between different feedback types for when the neuronal networks were engaged in *Gameplay*. Most notably, the finding that the open-loop *No-feedback* condition, where modulated activity from the culture was unable to affect the game outcome or alter the feedback received, showed considerably closer dynamics to criticality than when cultures were resting is interesting. This suggests that structured information input alone may be sufficient to induce these near-critical states in neuronal systems, however, information alone is insufficient in creating an evolving learning system as feedback is required as well. Furthermore, feedback does not necessarily need to be a positive addition to the system as identified in the experiments utilizing *Silent* feedback conditions. Taken in the context of attentional engagement and criticality, it is possible that facilitating an external source of information to impact the internal neural dynamics is necessary to drive these characteristics observed here and may also relate to in vivo results^45,46^. Indeed, a parallel may be made between groups of biological systems such as bird flocks and insect swarms, which have also been shown to exhibit criticality in movement patterns - presumably without a collective cognition. Yet in these cases, external information sources act upon the systems as a whole to shape the broader response, as in the case of the embodied neurons in this work.

Ultimately, here we found that by allowing cultures to be embodied and alter the environmental stimulation through action substantially pushes cultures closer to a critical dynamic compared to purely spontaneous activity. Not only does this showcase the utility of SBI systems for investigating these otherwise intractable questions but offers support for the idea of considering criticality as a spectrum. We propose that this work allows us to demonstrate one end of this spectrum, where criticality requires the input of structured information to a system to arise. This finding is entirely consistent with all rigorous studies into criticality, yet highlights - albeit with preliminary evidence - the importance of not relying solely on investigating criticality in a steady state of activity, such as spontaneous activity which is commonly done. Future work is still needed to further explain the more specific role of criticality in information processing and cognition - both in vivo and in vitro. This early work also helps provide an understanding of how work with embodied neural systems can be developed in an ethically appropriate manner by improving our understanding of these neurocomputational metrics and how they may or may not reflect given traits of interests^66,67^. Yet while questions about how neural criticality is linked with human cognition remain, ultimately, this work has suitably established that closeness to criticality appears as a fundamental property to neuronal assemblies, especially when influenced by the input of structured information in a closed-loop system. This provides additional compelling data to better understand the critical aspects of how our brains process information and may offer insight into more nuanced methods to understand this dynamic for future investigations.

## Methods

### Cell culture & MEA setup

Neural cells were cultured either from the cortices of E15.5 mouse embryos or differentiated from human induced pluripotent stem cells (hiPSCs) via a dual SMAD inhibition (DSI) protocol or through a lentivirus based NGN2 direct differentation protocols as previously described in ref. ^48^. MaxOne Multielectrode Arrays (MEA; Maxwell Biosystems, AG, Switzerland) were coated with either polyethylenimine (PEI) in borate buffer for primary culture cells or Poly-D-Lysine for cells from an iPSC background before being coated with either 10 *μ*g/ml mouse laminin or 10 *μ*g/ml human 521 Laminin (Stemcell Technologies Australia, Melbourne, Australia) respectively to facilitate cell adhesion. Approximately 10^6^ cells were plated on MEA after preparation as per ref. ^48^. Further details are described in Supplementary Information sections 1.1 & 1.2.

### *Dishbrain* platform and electrode configuration for input and output

The current *DishBrain* platform is configured as a low-latency, real-time MEA control system with on-line spike detection and recording software as described previously in ref. ^48^. Stimulation is applied in a topographically consistent manner across 8 electrodes for the relative position of the simulated ball to the simulated paddle in the simplified pong game. Counterbalanced pre-designated regions were defined, where greater activity across one set of regions would cause the simulated paddle to move in one direction, while greater activity in the other regions would prompt the paddle to move inversely. Additional stimulation input was delivered as feedback in response to the paddle either ’hitting’ or ’missing’ the simulated ball. These details are described fully in Supplementary Information sections 1.3–1.5.

### Data analysis

The avalanche analysis is performed in order to study the network in terms of its distance from criticality. The start and stop of an avalanche are determined by crossing a threshold of network activity^3^. An avalanche can be initiated by spikes from any and all neurons within a region of interest. The number of contributing spikes in each avalanche (*S*) and the total duration of the event (*D*) are then measured. To demonstrate the distance from criticality in a cultured cortical network, the presence of the following markers were investigated in the dynamics of our data: 1) Power law observables; 2) Exponent relation; 3) Branching ratio parameter; and 4) Scaling function. Certain criteria on these markers are the necessary conditions of a critical regime and meeting those criteria can indicate with a high confidence whether the system lies near a critical point^5,49^.

#### Power law observables

A critical system has interacting components (here, neurons) that show some fluctuation in their activity while also maintaining a level of correlation between their individual activities (here, individual spiking). Criticality implies that the system is defined by scale free dynamics and that events in both the spatial and temporal domains obey power laws^61,68,69^. For the networks considered here, events are contiguous cascades of spiking activity, rather than limited local bursts of spiking activity or huge network-wide spiking events. These contiguous cascades of spiking activity are called *neuronal avalanches*.

To investigate this property in our BNN system, binary spike trains of each neuron’s activity were utilized. The whole duration of each recording session was discretized to 50 ms bins. The sum of all cells’ activities in each time bin was used as the network activity. Next, a threshold of 40% of the median spiking activity in the network among all time bins was introduced. The start and end points of an avalanche were defined as the time points when the network activity crossed this threshold value from below and then above^53^. Our results were statistically robust across a range of activity thresholds between 30% and 70%. The size of an avalanche, *S*, is the total number of spikes during the avalanche. The avalanche duration, *D*, is the time between threshold crossings. Similar to^3^, maximum likelihood estimation was used to fit a truncated power law to the avalanche size distribution:

1 $$f(S)=\frac{{S}^{-\tau }}{\mathop{\sum }\nolimits_{{S}_{\min }}^{{S}_{\max }}{S}^{-\tau }},$$

where *τ* is the power law exponent corresponding to avalanche sizes. For a neuronal recording session in which *N*_*A*_ avalanches are detected, the fitting process to obtain the above equation is the following iterative procedure^70^:

1. Find the maximum observed avalanche size ${S}_{\max }$.
2. Evaluate the three different power law exponents, *τ*, for the 3 smallest avalanche sizes observed, ${S}_{\min }$.
3. Calculate the *Kolmogorov-Smirnov* (KS) test for this estimation to determine the goodness-of-fit between the fitted power law and the empirical distribution.
4. Among the obtained KS values, choose the smallest one, together with the corresponding *τ* and ${S}_{\min }$ values.
5. Complete the estimation if KS $< \frac{1}{\sqrt{{N}_{A}}}$ or otherwise repeat steps 2 to 5 with ${S}_{\max }$ reduced by 1 until this condition is met.

Steps 3 to 5 are necessary to ensure the data distribution indeed comes from a power law rather than another candidate heavy-tailed distribution, such as log normal and stretched exponential forms^71^. Applying the exact same procedure to the set of *D of the* avalanche events, the corresponding power law exponent of *α* was calculated for the entire avalanche duration distribution.

To test the validity of a power law fit to avalanche distributions, hypothesis testing was performed as described in ref. ^3^. For this purpose, the power law exponent, the number of detected avalanches, and the minimum and maximum avalanche sizes were set the same as the experimental avalanche distribution to generate 1000 artificial power law distributions. We generated these surrogate distributions using the inverse method as $S={S}_{min}{(1-r)}^{\frac{-1}{\tau -1}}$ where *r* was a random number sampled from a uniform distribution between 0 and 1. Then any surrogate distribution was upper-truncated at the maximum cut-off equivalent to *S*_*max*_ from the empirical data. The KS statistics was then employed to estimate the distance between the simulated surrogate distributions and a perfect power law. The *p* value determining the significance level was then equal to the ratio of the surrogate distributions with KS values smaller than the KS value of the corresponding experimental avalanche distribution. With significance level set to 0.05, *p* < 0.05 implies a rejection of the power law hypothesis while *p* ≥ 0.05 suggests the power law hypothesis was not rejected (the fit was good).

#### Exponent relation and deviation from criticality coefficient (DCC)

In critical systems, there is another exponent relationship between the power law parameters (*α* and *τ*) and the exponent of mean avalanche sizes (〈*S*〉), given their duration, *D*^72^. We first find this third power law exponent of the system, *β*, from the experimental data using linear regression given the following exponent relation is present in a critical system:

2 $$\langle S\rangle \propto {D}^{{\beta }_{{{{{{{{\rm{fit}}}}}}}}}}.$$

This third power law exponent also relates the size and duration distributions of the avalanches and is predicted by:

3 $${\beta }_{{{{{{{{\rm{pred}}}}}}}}}=\frac{(\alpha -1)}{(\tau -1)}.$$

Comparing the fitted value from the empirical data (*β*_fit_) and its estimation using *α* and *τ* exponents (*β*_pred_), a new measure is derived to evaluate the *Deviation from Criticality Coefficient* (DCC), parameterised as *d*_*CC*_:

4 $${d}_{CC}=\parallel {\beta }_{{{{{{{{\rm{pred}}}}}}}}}-{\beta }_{{{{{{{{\rm{fit}}}}}}}}}\parallel,$$

where *β*_pred_ and *β*_fit_ are the predicted and fitted values of *β* respectively. Consequently, a smaller DCC value indicates a more accurately fit power law distribution to the empirical data.

#### Branching ratio

The branching ratio is defined as the ratio of the number of units (neurons) active (spiking) at time step *t* + 1 to the number of active units (neurons) at time step *t*. Since a critical regime is naturally balanced and avoids runaway gains, the critical branching ratio is 1. Consequently, on average, network activity neither saturates nor dampens over time.

Suppose that *N* active neurons are detected in total and the number of active neurons in each time step *t* is defined by *N*(*t*). A fixed branching ratio of *m*, gives:

5 $$\langle N(t+1) \, | \, N(t)\rangle=mN(t)+h,$$

where 〈∣〉 is the conditional expectation and *h* is the mean rate of external drive. The activity decreases if *m* < 1, whereas it grows exponentially if *m* > 1, meaning that *m* = 1 separates these two regimens and represents a critical dynamic point. A precise prediction of *m* helps to assess the risk that *N*(*t*) will develop large and devastating avalanches of events such as epileptic seizures.

Under the circumstances when the full activity *N*(*t*) is known, *m* can be conventionally estimated using linear regression. Nevertheless, when using subsampling, when only a fraction of neurons in a neuronal network are sampled, this conventional method will be biased to some extent. The bias vanishes only if all units are sampled, because it is inherent to subsampling and cannot be overcome by obtaining longer recordings. Instead, inspired by the method introduced in^49^ the subsampled activity *n*(*t*) is utilized, where the fraction of recorded units to all cells is defined as a constant *μ*. *n*(*t*) here is a random variable whose expectation is proportional to the real *N*(*t*) and 〈*n*(*t*) ∣ *N*(*t*)〉 = *μN*(*t*) + *ξ*, where *μ* and *ξ* are constants. The bias value for the conventional linear estimator can now be calculated as:

6 $$m\left(\frac{{\mu }^{2}{{{{{{{\rm{var}}}}}}}}(N(t))}{{{{{{{{\rm{var}}}}}}}}(n(t))-1}\right).$$

To overcome this subsampling bias, the method introduced by Wilting and Priesemann^49^ was utilized. Instead of directly using the biased regression of activity at time *t* and *t* + 1, multiple linear regressions of activity between times *t* and *t* + *k* were performed with different time lags $k=1,\ldots,{k}_{\max }$. Each of these *k* values returns a regression coefficient *r*_*k*_ with *r*_1_ being equal to the result of a conventional estimator of *m*. With subsampling, all these regression slopes are biased by the same factor $b=\frac{{\mu }^{2}{{{{{{{\rm{var}}}}}}}}(N(t))}{{{{{{{{\rm{var}}}}}}}}(n(t))}$. In these circumstances, instead of the exponential relation *r*_*k*_ = *m*^*k*^ which is expected under full sampling, the equation generalizes to:

7 $${r}_{k}=\frac{{\mu }^{2}{{{{{{{\rm{var}}}}}}}}(N(t))}{{{{{{{{\rm{var}}}}}}}}(n(t))}.{m}^{k}=b.{m}^{k}.$$

Having multiple calculated *r*_*k*_ values, both *b* and *m* are estimated, which are constant for all *k*.

Figure S6 compares the estimated branching ratio parameter from 6 different cultured cortical networks during a *Gameplay* and a *Rest* session.

#### Scaling function

Another feature of critical dynamics is that avalanche shapes show fractal properties and all avalanche profiles of different sizes are scaled versions of the universal same shape. According to^72^, the value of *β* obtained from the exponent relation analysis can be used to calculate a scaling function for the avalanche shapes. For any given avalanche duration *D*, the average number of neurons firing at time *t* (within *D* seconds) is defined by *s*(*t*, *D*). The following relations hold in this system:

8 $$\begin{array}{rcl}s(t,\, D)&\propto &{D}^{\gamma }F\left(\frac{t}{D}\right)\\ \langle S\rangle (D)&=&\int\nolimits_{0}^{D}s(t,\, D)dt,\end{array}$$

where $F\left(\frac{t}{D}\right)$ is a universal function for all avalanches and *γ* = *β* − 1. Hence in this process, an initial *β* is used to predict *γ* and using this *γ* and the first term in Equation (8), $F\left(\frac{t}{D}\right)$ is obtained as $\langle \frac{s(t,D)}{{D}^{\gamma }}\rangle$. Here 〈.〉 denotes the average over all avalanches with duration *D*. A collection of $F\left(\frac{t}{D}\right)$ functions are extracted for various *D* durations. The error for this process is described as:

9 $$\frac{{{{{{{{\rm{var}}}}}}}}(F)}{{(\max (F)-\min (F))}^{2}}.$$

Repeating this process with various values for *β*, the exponent that produces the smallest error in Equation (9) is selected as the final scaling factor. In principle, we expect to obtain similar (if not the same) *β* values from this analysis and the estimates in Section 'Exponent relation and Deviation from Critically Coefficient (DCC)' near the critical point. We report the difference between these two *β* values as the SC error. The NCC toolbox in MATLAB^50^ was utilized to perform shape collapse on data. This shape collapse error is expected to be minimized under critical conditions. For shape collapse, avalanches with durations from 4 to 20 bins (200 to 1000 ms) were considered. Across the time course of our recordings, there were not enough avalanches to conduct meaningful shape collapse analysis, beyond these cutoffs.

The schematic in Figure S7 summarizes the main attributes of a near-critical system compared to super/sub-critical states.

### Burst pattern analysis

Inspired by the methods and metrics for classification of bursts (or avalanches) introduced in ref. ^52^, we extracted the following quantitative details from the *Rest* state recordings of each in vitro culture which were applicable to our dataset. These measurements were then used as classifiers to distinguish between cultures based on their bursting patterns and finally to identify whether any of the criticality metrics studied in our paper showed a significant difference between the classes of cultures with different bursting behaviors. The 1) Size distribution of bursts (or avalanches), 2) Burst Rates of avalanches, and 3) Superbursts were the quantitative criteria extracted from all of the rest state spontaneous activity of the cultures to classify them.

Below is a brief explanation of each calculated criteria:

- Size distribution: Within some recordings, bursts exhibited highly similar sizes, while in others a broad range of burst sizes was observed. The range of burst sizes varied among recordings, with some displaying a continuum of sizes and others having distinct clusters of large and small bursts with very few bursts of intermediate size. In every recording from a specific culture, let N* denote the number of spikes in the third-largest burst. Bursts containing at least 75% of N* spikes were classified as large, while those with at least 25% but less than 75% of N* spikes were classified as medium. Bursts containing fewer than 25% of N* spikes were labeled as small. If the number of medium bursts exceeded the number of large bursts, the burst size was considered ‘*variable*’ or *‘irregular’*. Conversely, if the number of small bursts exceeded the number of large bursts, the burst size distribution was considered *‘bimodal’*. If the number of large bursts exceeded that of medium or small bursts, the burst size was deemed *‘fixed’*. To avoid confusion with the terms employed to classify distinct forms of bursting rates, we chose to use the term *‘irregular’* instead of the original term (‘variable’) proposed by^52^.
- Burst Rates: The categorization of burst patterns was further expanded based on their burst rates, which were usually consistent over time and could exhibit either regular or more chaotic intervals. A burst rate was considered *‘highly variable’* if the maximum rate, calculated from the shortest time interval that contained 10 inter-burst intervals, differed by a factor of 10 or more from the minimum rate, calculated from the longest time interval that contained only 3 inter-burst intervals. Otherwise, the burst rate was classified as *‘non-variable’*.
- Superbursts: A recording was classified as being dominated by superbursts if at least half of all large and medium bursts occurred within tightly clustered intervals, where the inter-cluster intervals were at least 10 times longer than the intra-cluster intervals. Superbursts were categorized as *‘regular’* if the variance of the number of bursts per superburst was small, i.e. less than half of the average. If the variance exceeded this threshold, the superbursts were classified as *‘short’* if the mean number of bursts per superburst was less than 10, or *‘long’* otherwise.

### Reporting summary

Further information on research design is available in the Nature Portfolio Reporting Summary linked to this article.

### Supplementary information

Supplementary Information

Reporting Summary

### Source data

Source Data

## Data Availability

All data generated for or used within this manuscript have been deposited at Open Science Framework (OSF) and are publicly available here: https://osf.io/ncvpq/?view_only=8fc5fc5aad254fce92a79390ae84b81c. Source data are provided with this paper.

## Code availability

All code for data analysis to generate the results in this manuscript have been deposited at Open Science Framework (OSF) and are publicly available via https://osf.io/ncvpq/?view_only=8fc5fc5aad254fce92a79390ae84b81c.
