# Supplementary material for: Critical dynamics arise during structured information presentation within embodied in vitro neuronal networks
Source: Nat Commun. 2023 Aug 30;14:5287. doi: 10.1038/s41467-023-41020-3 (PMC10469171; doi:10.1038/s41467-023-41020-3)
Supplement: Supplementary file 1 — Supplementary Information [file 41467_2023_41020_MOESM1_ESM.pdf]

Critical dynamics arise during structured information presentation within embodied *in vitro* neuronal networks

Forough Habibollahi[1,2,3†], Brett J. Kagan[2*†], Anthony N. Burkitt[1] and Chris French[3]

[1]Biomedical Engineering Department, University of Melbourne, Parkville, 3010, VIC, Australia.
[2]Cortical Labs Pty Ltd, Melbourne, 3056, VIC, Australia.
[3]Neural Dynamics Laboratory, Department of Medicine, University of Melbourne, Parkville, 3010, VIC, Australia.

*Corresponding author(s). E-mail(s): brett@corticallabs.com;
†These authors contributed equally to this work.

Supplementary Information

1 Supplementary Methods

1.1 Cell Culture

Neural cells were cultured either from the cortices of E15.5 mouse embryos or differentiated from human induced pluripotent stem cells (hiPSCs) via a dual SMAD inhibition (DSI) protocol or through a lentivirus based NGN2 direct differentation protocols as previously described [1]. Mice were BL6/C57 stains and were mated at Monash Animal Research Platform (MARP). Upon confirmation of pregnancy, animals were transported via an approved carrier to the Alfred Medical Research and Education Precinct (AMREP). Pregnant animals were housed in individually ventilated cages until the date when they were humanely killed, and primary cells were harvested. The hiPSC line was RM3.5 GT-GFP-01 constitutively expressing green fluorescent protein under the GAPDH promoter was utilised. The RM3.5 hiPSC line is from an XY donor and was initially derived from human foreskin fibroblasts and reprogrammed using the hSTEMCCAloxP four factor lentiviral vector as reported and validated previously [2]. Cells were cultured until plating. For primary mouse neurons this occurred at day-in-vitro (DIV) 0, for DSI cultures this occurred at between DIV 30 - 33 depending culture development, for NGN2 cultures this occured at DIV 3.

1.2 MEA Setup and Plating

MaxOne Multielectrode Arrays (MEA; Maxwell Biosystems, AG, Switzerland) was used and is a high-resolution electrophysiology platform featuring 26,000 platinum electrodes arranged over an $8mm^2$ surface. The MaxOne system is based on complementary meta-oxide-semiconductor (CMOS) technology and allows recording from up to 1024 channels. MEAs were coated with either polyethylenimine (PEI) in borate buffer for primary culture cells or Poly-D-Lysine for cells from an iPSC background before being coated with either 10 µg/ml mouse laminin or 10 µg/ml human 521 Laminin (Stemcell Technologies Australia, Melbourne, Australia) respectively to facilitate cell adhesion. Approximately 10^6 cells were plated on MEA after preparation as per [1]. Cells were allowed approximately one hour to adhere to MEA surface before the well was flooded. The day after plating, cell culture media was changed for all culture types to BrainPhys™ Neuronal Medium (Stemcell Technologies Australia, Melbourne, Australia) supplemented with 1% penicillin-streptomycin. Cultures were maintained in a low O2 incubator kept at 5% CO2, 5% O2, 36°C and 80% relative humidity. Every two days, half the media from each well was removed and replaced with free media. Media changes always occurred after all recording sessions.

1.3 Dishbrain platform and electrode configuration

The current DishBrain platform is configured as a low-latency, real-time MEA control system with on-line spike detection and recording software. The DishBrain platform provides on-line spike detection and recording configured as a low-latency, real-time MEA control. The DishBrain software runs at 20 kHz and allows recording at an incredibly fine timescale. There is the option of recording spikes in binary files, and regardless of recording, they are counted over a period of 10 milliseconds (200 samples), at which point the game environment is provided with how many spikes are detected in each electrode in each predefined motor region as described below. Based on which motor region the spikes occurred in, they are interpreted as motor activity, moving the 'paddle' up or down in the virtual space. As the ball moves around the play area at a fixed speed and bounces off the edge of the play area and the paddle, the 'Pong' game is also updated at every 10ms interval. Once the ball hits the edge of the play area behind the paddle, one rally of 'Pong' has come to an end. The game environment will instead determine which type of feedback to apply at the end of the rally: random, silent, or none. Feedback is also provided when the ball contacts the paddle under the standard stimulus condition. A 'stimulation sequencer' module tracks the location of the ball relative to the paddle during each rally and encodes it as stimulation to one of eight stimulation sites. Each time a sample is received from the MEA, the stimulation sequencer is updated 20,000 times a second, and after the previous lot of MEA commands has completed, it constructs a new sequence of MEA commands based on the information it has been configured to transmit based on both place codes and rate codes. The stimulations take the form of a short square bi-phasic pulse that is a positive voltage, then a negative voltage. This pulse sequence is read and applied to the electrode by a Digital to Analog Converter (or DAC) on the MEA. A real-time interactive version of the game visualiseris available at https://spikestream.corticallabs.com/. Alternatively, cells could be recorded at 'rest' in a gameplay environment where activity was recorded to move the paddle but no stimulation was delivered, with corresponding outcomes still recorded. Using this spontaneous activity alone as a baseline, the gameplay characteristics of a culture were determined. Low level code for interacting with Maxwell API was written in C to minimize processing latencies-so packet processing latency was typically $<50\ \mu$s. High-level code was written in Python, including configuration setups and general instructions for game settings. A 5 ms spike-to-stim latency was achieved, which was substantially due to MaxOne's inflexible hardware buffering. Figure S1 illustrates a schematic view of Software components and data flow in the DishBrain closed loop system.

1.4 Input Configuration

As introduced in [1], stimulation is delivered at specific locations, frequency, and voltage to key electrodes in a topographically consistent manner in the sensory area relative to the current position of the paddle (see Figure S2). This

mimics retinotopic and topographic representations found in many neural systems, which represent the external world [3, 4]. It was possible to deliver five types of input. Either the 'Sensory Stimulus' encoding the position of the ball, or one of four feedback protocols explained below: Unpredictable, Predictable, Silent, or No-feedback.

Sensory Stimulus: Due to the fact that neurons appeared robust to voltage stimulation, the voltage level was determined based on the evidence of neurological function. As a result, 75 mV was chosen as the sensory stimulation voltage to prevent forcing hyperpolarised cells to fire. This stimulation was applied to key electrodes relating to where the ball was relative to the paddle. Combining place coding with a rate coding, when the ball was closest to the opposing wall, stimuli were delivered at 4 Hz, increasing in a linear manner to 40 Hz when it reached the paddle wall.

Unpredictable Stimulus: For the standard stimulus feedback condition, cultures received unpredictable stimulation when they missed connecting the paddle with the 'ball', i.e. when a 'miss' occurred. Using a feedback stimulus at a voltage of 150 mV and a frequency of 5 Hz, unpredictable external stimulus could be added to the system. Random stimulation took place at random sites over the 8 predefined input electrodes at random timescales for a period of four seconds, followed by a configurable rest period of four seconds where stimulation paused, then the next rally began. In theory, the higher voltage than used for the Sensory Stimulus would force action potentials regardless of the state in which the cell was in, causing even greater disruption.

Predictable Stimulus: For the standard stimulus feedback condition, cultures were exposed to predictable stimulation when a 'hit' was registered - that is, when the 'paddle' connected successfully with the 'ball'. This was delivered at 75mV at 100Hz over 100ms. This occurred when the simulated ball struck the paddle and replaced other sensory information for 100 ms. All 8 stimulation electrodes simultaneously received predictable stimulation at this frequency and period.

Silent Feedback: During Silent feedback period, the Unpredictable Stimulus described above was replaced with no stimulation for the same length of time. Predictable Stimulus feedback was also removed during Silent Feedback sessions. There is still a difference between this feedback and No-Feedback described below. This feedback is associated with culture activity in a closed-loop manner and therefore constitutes feedback.

No-feedback: As an open-loop condition, this assessment was designed to determine whether sensory stimulation is sufficient to drive learning in cultures. In other words, there was no feedback of any kind provided to the cultures based on their actions or outcomes. As described above, the cultures were given the same sensory stimulation as described above, and the outcome was determined by the same metric. However, when a 'miss' would otherwise occur, here instead the ball bounced off the wall behind the paddle, continuing the same trajectory – still recorded as a 'miss'. This would otherwise end the rally. The 'ball' would be recorded as a 'hit' whenever it connected with

the simulated paddle. So, under No-Feedback, the entire gameplay session is essentially one rally in which the simulated ball's final position can be predicted from its initial vector, but scoring occurs normally as usual.
Figure S3 represents a schematic showing the different phases of stimulation and the information delivery to the culture while Figure S4 demonstrates different simulated gameplay environments employing different feedback protocols as above.

1.5 Output Configuration

A total of 1024 electrodes were routed on the HD-MEA to record activity. The 'Sensory' area, where stimulation electrodes were embedded as described above consisted of 626 electrodes. The remaining output electrodes were divided into predefined motor regionson the MEA, consisting of four regions that were defined either as motor region 1 or motor region 2 as shown in Figure S2. Since it was technically difficult to cultivate neurons that displayed perfectly symmetrical activity in both these regions, 'gain' was added to the system. These took a real-time value based on the mean firing in each motor region and multiplied it to achieve a target value of 20 Hz across the entire region. Consequently, changes in activity in each of the two regions could affect the position of the paddle, even if they displayed different latent spontaneous activity.

1.6 Culture and experiment statistics

For a combination of reasons there were differences in the number of experiments each culture performed. Tables in Figure S5.a-d represent the details of the number of *Gameplay* sessions performed by each culture in each condition and the corresponding dates of the experiments.

2 Supplementary Figures

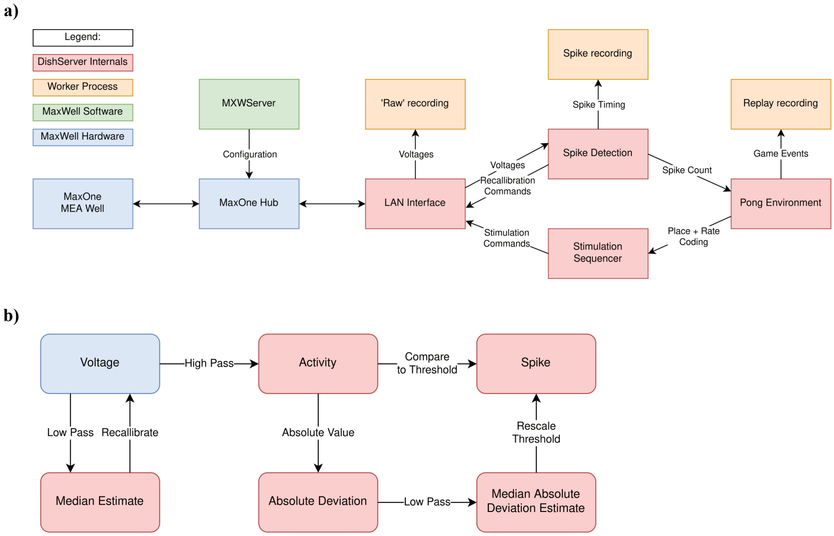

Fig. S1 **a, b)** Schematics of software used for DishBrain. **a)** Software components and data flow in the DishBrain closed loop system. Voltage samples flow from the MEA to the ‘Pong’ environment, and sensory information flows from the ‘Pong’ environment back to the MEA, forming a closed loop. The blue rectangles mark proprietary pieces of hardware from MaxWell, including the MEA well which may contain a live culture of neurons. The green MXWServer is a piece of software provided by MaxWell which is used to configure the MEA and Hub, using a private API directly over the network. The red rectangles mark components of the ‘DishServer’ program, a high-performance program consisting of four components designed to run asynchronously, despite being run on a single CPU thread. The ‘LAN Interface’ component stores network state, for talking to the Hub, and produces arrays of voltage values for processing. Voltage values are passed to the ‘Spike Detection’ component, which stores feedback values and spike counts, and passes recalibration commands back to the LAN Interface. When the ‘Pong’ environment is ready to run, it updates the state of the paddle based on the spike counts, the state of the ball based on its velocity and collision conditions, and reconfigures the stimulation sequencer based on the relative position of the ball and current state of the game. The stimulation sequencer stores and updates indices and countdowns relating to the stimulations it must produce and converts these into commands each time the corresponding countdown reaches zero, which are finally passed back to the LAN Interface, to send to the MEA system, closing the loop. The procedures associated with each component are run one after the other in a simple loop control flow, but the ‘Pong’ environment only moves forward every 200th update, short-circuiting otherwise. Additionally, up to three worker processes are launched in parallel, depending on which parts of the system need to be recorded. They receive data from the main thread via shared memory and write it to file, allowing the main thread to continue processing data without having to hand control to the operating system and back again. **b)** Numeric operations in the real-time spike detection component of the DishBrain closed loop system, including multiple IIR filters. Running a virtual environment in a closed loop imposes strict performance requirements, and digital signal processing is the main bottleneck of this system, with close to 42 MB of data to process every second. Simple sequences of IIR digital filters is applied to incoming data, storing multiple arrays of 1024 feedback values in between each sample. First, spikes on the incoming data are detected by a high pass filter to determine the deviation of the activity, and comparing that to the MAD, which is itself calculated with a subsequent low pass filter. Then, a low pass filter is used to the original data to determine whether the MEA hardware needs to be re-calibrated, affecting future samples. This system was able to keep up with the incoming data on a single thread of an Intel Core i7-8809G. Figures adapted from [1]. This article was published in Neuron, 110.23, Kagan, Brett J., Andy C. Kitchen, Nhi T. Tran, Forough Habibollahi, Moein Khajehnejad, Bradyn J. Parker, Anjali Bhat, Ben Rollo, Adeel Razi, and Karl J. Friston., ”In vitro neurons learn and exhibit sentience when embodied in a simulated game-world.”, 3952-3969, Copyright The Author(s). Published by Elsevier Inc. (2022).

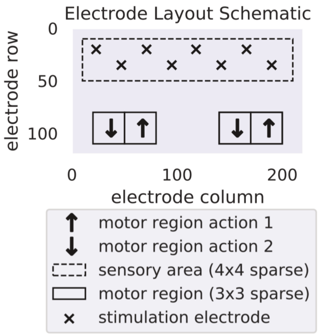

Fig. S2 Electrode layout schematic for DishBrain Pong-world gameplay. Figure adapted from [1]. This article was published in Neuron, 110.23, Kagan, Brett J., Andy C. Kitchen, Nhi T. Tran, Forough Habibollahi, Moein Khajehnejad, Bradyn J. Parker, Anjali Bhat, Ben Rollo, Adeel Razi, and Karl J. Friston., "In vitro neurons learn and exhibit sentience when embodied in a simulated game-world.", 3952-3969, Copyright The Author(s). Published by Elsevier Inc. (2022).

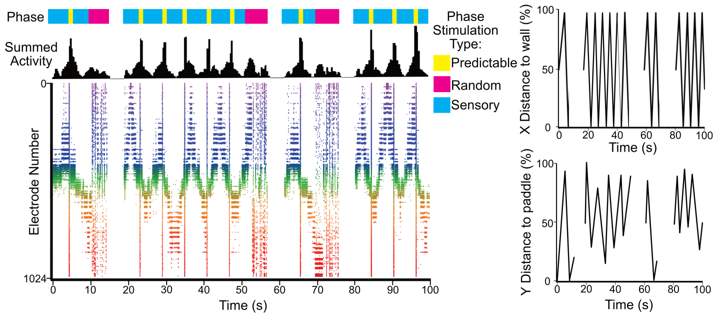

Fig. S3 Presents a schematic showing the different phases of stimulation which provides information about the environment to the culture, in line with this is the corresponding input voltage and how that voltage appears on the raster plot over 100 seconds. The appearance of random stimulation after a ball missing vs system wide predictable stimulation upon a successful hit is apparent across all three representations. This corresponds to the images on the right which show the position of the ball on both x and y axis relative to the paddle and backwall in % of total distance is shown on the same timescale. Figure adapted from [1]. This article was published in Neuron, 110.23, Kagan, Brett J., Andy C. Kitchen, Nhi T. Tran, Forough Habibollahi, Moein Khajehnejad, Bradyn J. Parker, Anjali Bhat, Ben Rollo, Adeel Razi, and Karl J. Friston., "In vitro neurons learn and exhibit sentience when embodied in a simulated game-world.", 3952-3969, Copyright The Author(s). Published by Elsevier Inc. (2022).

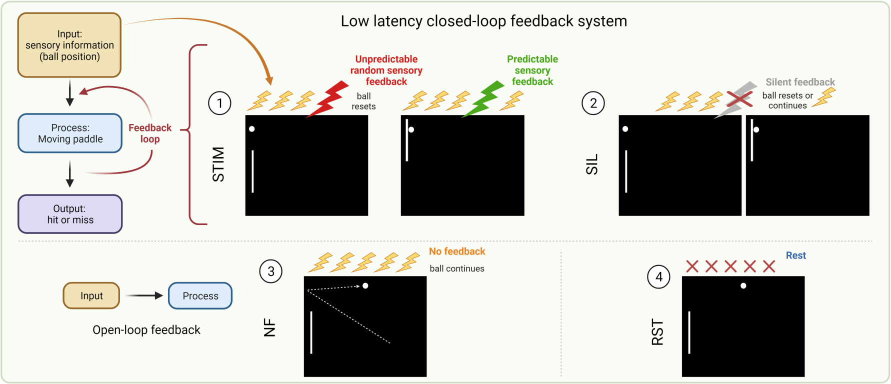

Fig. S4 Different DishBrain environments were utilised to demonstrate: (1&2) low latency closed-loop feedback system (stimulation (STIM) & silent (SIL) treatment); (3) No feedback (NF) system to demonstrate an open-loop feedback configuration; and (4) rest (RST) configuration to demonstrate a system in which sensory information (yellow bolt) is absent. Figure adapted from [1]. This article was published in Neuron, 110.23, Kagan, Brett J., Andy C. Kitchen, Nhi T. Tran, Forough Habibollahi, Moein Khajehnejad, Bradyn J. Parker, Anjali Bhat, Ben Rollo, Adeel Razi, and Karl J. Friston., "In vitro neurons learn and exhibit sentience when embodied in a simulated game-world.", 3952-3969, Copyright The Author(s). Published by Elsevier Inc. (2022).

a)

Original Experiments

Culture ID	# of Gameplay Experiments	Experiment date: # of Gameplay recordings
9323	3	{'21-04-23': 3}
11615	6	{'21-06-18': 3, '21-06-21': 3}
9468	6	{'21-06-18': 3, '21-06-21': 3}
7282	7	{'21-02-22': 2, '21-02-23': 2, '21-02-19': 2, '21-03-01': 1}
11621	9	{'21-05-03': 3, '21-05-31': 3, '21-06-01': 2, '21-06-02': 1}
9431	10	{'21-04-21': 3, '21-04-23': 3, '21-04-27': 2, '21-04-22': 2}
11619	11	{'21-06-02': 3, '21-05-31': 2, '21-05-03': 2, '21-05-04': 2, '21-06-01': 2}
9501	12	{'21-04-16': 3, '21-05-31': 3, '21-06-01': 3, '21-06-02': 3}
11614	15	{'21-05-03': 3, '21-05-04': 3, '21-05-31': 3, '21-06-01': 3, '21-06-02': 3}
9353	16	{'21-06-10': 3, '21-06-11': 3, '21-06-17': 3, '21-06-07': 3, '21-06-02': 2, '21-06-18': 2}
9501i	16	{'21-04-20': 3, '21-04-21': 3, '21-04-22': 3, '21-04-27': 3, '21-04-23': 2, '21-04-19': 2}
9358	26	{'21-06-16': 3, '21-06-17': 3, '21-06-18': 3, '21-06-15': 3, '21-06-11': 3, '21-06-09': 3, '21-06-07': 3, '21-06-02': 3, '21-06-10': 2}
9495	26	{'21-06-02': 3, '21-06-07': 3, '21-06-09': 3, '21-06-10': 3, '21-06-11': 3, '21-06-15': 3, '21-06-17': 3, '21-06-18': 3, '21-06-16': 2}
9381	29	{'21-04-16': 3, '21-04-19': 3, '21-04-21': 3, '21-04-22': 3, '21-04-23': 3, '21-04-27': 3, '21-05-31': 3, '21-06-01': 3, '21-06-02': 3, '21-04-20': 2}

b)

Stimulus Feedback

Culture ID	# of Gameplay Experiments	Experiment date: # of Gameplay recordings
12106	3	{'2021-06-23': 3}
12160	3	{'2021-06-22': 3}
11609	8	{'2021-06-23': 3, '2021-06-25': 3, '2021-06-24': 2}
11570	9	{'2021-06-23': 3, '2021-06-24': 3, '2021-06-25': 3}
11614	9	{'2021-05-31': 3, '2021-06-01': 3, '2021-06-02': 3}
11619	9	{'2021-05-31': 3, '2021-06-01': 3, '2021-06-02': 3}
11621	9	{'2021-05-31': 3, '2021-06-01': 3, '2021-06-02': 3}
12123	9	{'2021-06-23': 3, '2021-06-22': 3, '2021-06-21': 3}
12138	9	{'2021-06-23': 3, '2021-06-22': 3, '2021-06-21': 3}
12250	9	{'2021-06-21': 3, '2021-06-22': 3, '2021-06-23': 3}
12265	9	{'2021-06-23': 3, '2021-06-21': 3, '2021-06-22': 3}
12285	9	{'2021-06-21': 3, '2021-06-22': 3, '2021-06-23': 3}
9381	9	{'2021-05-31': 3, '2021-06-01': 3, '2021-06-02': 3}
9501	9	{'2021-05-31': 3, '2021-06-01': 3, '2021-06-02': 3}

c)

Silent Feedback

Culture ID	# of Gameplay Experiments	Experiment date: # of Gameplay recordings
12265I	3	{'2021-06-24': 3}
3258	3	{'2021-06-22': 3}
9353	3	{'2021-06-22': 3}
12250	6	{'2021-06-29': 3, '2021-06-28': 3}
9358	6	{'2021-06-23': 3, '2021-06-24': 3}
9468	6	{'2021-06-22': 3, '2021-06-23': 3}
11570	9	{'2021-06-28': 3, '2021-06-29': 3, '2021-06-30': 3}
11609	9	{'2021-06-28': 3, '2021-06-29': 3, '2021-06-30': 3}
11615	9	{'2021-06-22': 3, '2021-06-23': 3, '2021-06-24': 3}
12265	9	{'2021-06-25': 3, '2021-06-28': 3, '2021-06-29': 3}
9495	9	{'2021-06-22': 3, '2021-06-23': 3, '2021-06-24': 3}
12138	11	{'2021-06-29': 3, '2021-06-25': 3, '2021-06-24': 3, '2021-06-28': 2}
12106	12	{'2021-06-27': 3, '2021-06-24': 3, '2021-06-28': 3, '2021-06-29': 3}
12123	12	{'2021-06-24': 3, '2021-06-27': 3, '2021-06-28': 3, '2021-06-29': 3}
12285	12	{'2021-06-24': 3, '2021-06-27': 3, '2021-06-28': 3, '2021-06-29': 3}

d)

No-Feedback

Culture ID	# of Gameplay Experiments	Experiment date: # of Gameplay recordings
13117	3	{'2021-07-02': 3}
13113	6	{'2021-07-03': 3, '2021-07-02': 3}
13168	6	{'2021-07-02': 3, '2021-07-03': 3}
12106	8	{'2021-07-03': 3, '2021-07-01': 3, '2021-07-02': 2}
11570	9	{'2021-07-01': 3, '2021-07-02': 3, '2021-07-03': 3}
11609	9	{'2021-07-03': 3, '2021-07-02': 3, '2021-07-01': 3}
11610	9	{'2021-07-03': 3, '2021-07-02': 3, '2021-07-01': 3}
12123	9	{'2021-07-03': 3, '2021-07-02': 3, '2021-07-01': 3}
12138	9	{'2021-07-03': 3, '2021-07-02': 3, '2021-07-01': 3}
12250	9	{'2021-07-03': 3, '2021-07-02': 3, '2021-07-01': 3}
12265	9	{'2021-07-03': 3, '2021-07-02': 3, '2021-07-01': 3}
12285	9	{'2021-07-03': 3, '2021-07-02': 3, '2021-07-01': 3}

Fig. S5 Number of *Gameplay* experiments and corresponding dates of experiments for **a)** the original recordings, **b)** the *Stimulus* condition, **c)** the *Silent* condition, and **d)** the *No-feedback* condition.

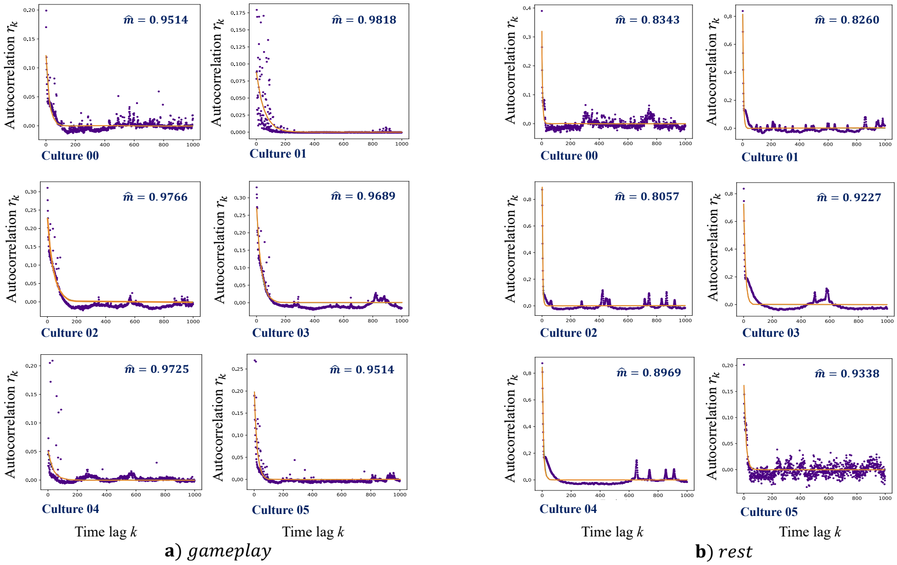

Fig. S6 Estimated branching ratio parameters ($\hat{m}$) measured using the method introduced in [7] for 6 sample recorded cultures during a **a)** ***Gameplay*** session followed by a **b)** ***Rest*** session.

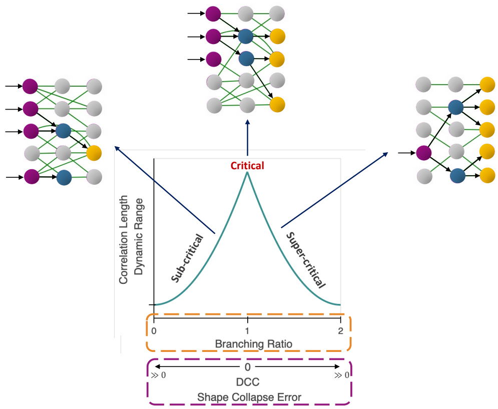

Fig. S7 Key characteristics of a critical system compared with *sub-critical* and *super-critical* dynamics. If tuned near criticality, the Branching Ratio tends to 1 whereas the DCC and Shape Collapse error are close to 0.

Table S1. Multivariate statistical tests and all results for tests done.

Figure	Panel	Parameter	Source	DF 1	DF2	MS	F	p-value	np2	Method
**4**	c	DCC	State (Gameplay/Rest)	1	294	8.808	33.959	0.000	0.104	Two-way Mixed ANOVA
			Region (motor/sensory)	1	294	0.012	-0.083	1.000	-0.000	
			Interaction	1	294	0.298	-2.048	1.000	-0.007	
	d	Branching Ratio	State (Gameplay/Rest)	1	296	0.255	21.224	0.000	0.067	Two-way Mixed ANOVA
			Region (motor/sensory)	1	296	0.001	-0.150	1.000	-0.001	
			Interaction	1	296	0.009	-1.356	1.000	-0.005	
	e	Shape Collapse Error	State (Gameplay/Rest)	1	298	11.090	12.260	0.001	0.040	Two-way Mixed ANOVA
			Region (motor/sensory)	1	298	0.152	-0.322	1.000	-0.001	
			Interaction	1	298	-0.037	0.078	0.780	0.000	
**5**	a	DCC	State (Gameplay/Rest)	1	199	5.759	25.407	0.000	0.113	Two-way Mixed ANOVA
			Size Distribution (bimodal/irregular)	1	199	1.018	-7.570	1.000	-0.039	
			Interaction	1	199	0.324	-2.411	1.000	-0.012	
	b	Branching Ratio	State (Gameplay/Rest)	1	199	0.245	16.949	0.000	0.078	Two-way Mixed ANOVA
			Size Distribution (bimodal/irregular)	1	199	0.052	-6.552	1.000	-0.034	
			Interaction	1	199	-0.017	2.100	0.149	0.010	
	c	Shape Collapse Error	State (Gameplay/Rest)	1	199	8.703	8.965	0.003	0.043	Two-way Mixed ANOVA
			Size Distribution (bimodal/irregular)	1	199	16.342	-27.698	1.000	-0.162	
			Interaction	1	199	0.128	-0.217	1.000	-0.001	
	d	DCC	State (Gameplay/Rest)	1	220	5.759	28.088	0.000	0.113	Two-way Mixed ANOVA
			Burst Rates (highly/not variable)	1	220	0.429	-3.680	1.000	-0.017	
			Interaction	1	220	-0.186	1.593	0.208	0.007	
	e	Branching Ratio	State (Gameplay/Rest)	1	220	0.245	18.738	0.0	0.078	Two-way Mixed ANOVA
			Burst Rates (highly/not variable)	1	220	0.003	-0.439	1.0	-0.002	
			Interaction	1	220	0.016	-2.232	1.0	-0.010	
	f	Shape Collapse Error	State (Gameplay/Rest)	1	220	8.703	9.911	0.002	0.043	Two-way Mixed ANOVA
			Burst Rates (highly/not variable)	1	220	13.246 189	-25.754	1.000	-0.133	
			Interaction	1	220	-1.036	2.014	0.157	0.009	

Table S2. Follow up main text post-hoc tests for multivariate tests, including means, standard error (SE), t-scores, degree of freedom and exact p-values with hedges.

Figure	Panel	Parameters	Source	A	B	mean (A)	mean(B)	diff	se	T	p-tukey	hedges	Method
**4**	g	DCC	Gameplay Status and Feedback Types	No-feedback	Rest	0.413	2.011	-1.597	0.266	-6.011	0.001	-0.808	Tukey's
				No-feedback	Silent	0.413	0.218	0.195	0.291	0.671	0.900	0.099	
				No-feedback	Stimulus	0.413	0.312	0.101	0.298	0.340	0.900	0.051	
				Rest	Silent	2.011	0.218	1.793	0.228	7.859	0.001	0.908	
				Rest	Stimulus	2.011	0.312	1.699	0.236	7.189	0.001	0.860	
				Silent	Stimulus	0.218	0.312	-0.094	0.265	-0.356	0.900	-0.048	

Supplementary References

[1] Kagan, B. J. *et al.* In vitro neurons learn and exhibit sentience when embodied in a simulated game-world. *Neuron* (2022) .

[2] Wilting, J. & Priesemann, V. Inferring collective dynamical states from widely unobserved systems. *Nature communications* **9** (1), 1–7 (2018) .

[3] Shlens, J. *et al.* The structure of multi-neuron firing patterns in primate retina. *Journal of Neuroscience* **26** (32), 8254–8266 (2006) .

[4] Baranes, K., Chejanovsky, N., Alon, N., Sharoni, A. & Shefi, O. Topographic cues of nano-scale height direct neuronal growth pattern. *Biotechnology and bioengineering* **109** (7), 1791–1797 (2012) .

[5] Ma, Z., Turrigiano, G. G., Wessel, R. & Hengen, K. B. Cortical circuit dynamics are homeostatically tuned to criticality in vivo. *Neuron* **104** (4), 655–664 (2019) .

[6] Beggs, J. M. & Plenz, D. Neuronal avalanches in neocortical circuits. *Journal of neuroscience* **23** (35), 11167–11177 (2003) .

[7] Wilting, J. & Priesemann, V. Inferring collective dynamical states from widely unobserved systems. *Nature communications* **9** (1), 1–7 (2018) .

[8] Beggs, J. M. & Timme, N. Being critical of criticality in the brain. *Frontiers in physiology* **3**, 163 (2012) .

[9] Yeomans, J. M. *Statistical mechanics of phase transitions* (Clarendon Press, 1992).

[10] Nishimori, H. & Ortiz, G. *Elements of phase transitions and critical phenomena* (Oup Oxford, 2010).

[11] Klaus, A., Yu, S. & Plenz, D. Statistical analyses support power law distributions found in neuronal avalanches. *PloS one* **6** (5), e19779 (2011) .

[12] Clauset, A., Shalizi, C. R. & Newman, M. E. Power-law distributions in empirical data. *SIAM review* **51** (4), 661–703 (2009) .

[13] Friedman, N. *et al.* Universal critical dynamics in high resolution neuronal avalanche data. *Physical review letters* **108** (20), 208102 (2012) .

[14] Marshall, N. *et al.* Analysis of power laws, shape collapses, and neural complexity: new techniques and matlab support via the ncc toolbox. *Frontiers in physiology* **7**, 250 (2016) .

Springer Nature 2021 LaTeX template

[15] Wagenaar, D. A., Pine, J. & Potter, S. M. An extremely rich repertoire of bursting patterns during the development of cortical cultures. *BMC neuroscience* **7** (1), 1–18 (2006) .
