# Supplementary material for: Critical dynamics arise during structured information presentation within embodied in vitro neuronal networks
Source: Nat Commun. 2023 Aug 30;14:5287. doi: 10.1038/s41467-023-41020-3 (PMC10469171; doi:10.1038/s41467-023-41020-3)
Supplement: Supplementary file 2 — Reporting Summary [file 41467_2023_41020_MOESM2_ESM.pdf]

nature portfolio

Corresponding author(s): Kagan

Last updated by author(s): 19/07/2023

Reporting Summary

Nature Portfolio wishes to improve the reproducibility of the work that we publish. This form provides structure for consistency and transparency in reporting. For further information on Nature Portfolio policies, see our Editorial Policies and the Editorial Policy Checklist.

Statistics

For all statistical analyses, confirm that the following items are present in the figure legend, table legend, main text, or Methods section.

n/a	Confirmed	
☐	☒	The exact sample size (*n*) for each experimental group/condition, given as a discrete number and unit of measurement
☐	☒	A statement on whether measurements were taken from distinct samples or whether the same sample was measured repeatedly
☐	☒	The statistical test(s) used AND whether they are one- or two-sided<br>*Only common tests should be described solely by name; describe more complex techniques in the Methods section.*
☐	☒	A description of all covariates tested
☐	☒	A description of any assumptions or corrections, such as tests of normality and adjustment for multiple comparisons
☐	☒	A full description of the statistical parameters including central tendency (e.g. means) or other basic estimates (e.g. regression coefficient) AND variation (e.g. standard deviation) or associated estimates of uncertainty (e.g. confidence intervals)
☐	☒	For null hypothesis testing, the test statistic (e.g. *F*, *t*, *r*) with confidence intervals, effect sizes, degrees of freedom and *P* value noted<br>*Give P values as exact values whenever suitable.*
☒	☐	For Bayesian analysis, information on the choice of priors and Markov chain Monte Carlo settings
☒	☐	For hierarchical and complex designs, identification of the appropriate level for tests and full reporting of outcomes
☐	☒	Estimates of effect sizes (e.g. Cohen's *d*, Pearson's *r*), indicating how they were calculated

Our web collection on statistics for biologists contains articles on many of the points above.

Software and code

Policy information about availability of computer code

Data collection	All data generated for or used within this manuscript have been deposited at Open Science Framework (OSF) and are publicly available here: https://osf.io/ncvpq/?view_only=8fc5fc5aad254fce92a79390ae84b81c
Data analysis	Custom codes generated in Python 3.9.6 and Matlab r2022b are available at: https://osf.io/ncvpq/?view_only=8fc5fc5aad254fce92a79390ae84b81c.

For manuscripts utilizing custom algorithms or software that are central to the research but not yet described in published literature, software must be made available to editors and reviewers. We strongly encourage code deposition in a community repository (e.g. GitHub). See the Nature Portfolio guidelines for submitting code & software for further information.

Data

Policy information about availability of data

All manuscripts must include a data availability statement. This statement should provide the following information, where applicable:

- Accession codes, unique identifiers, or web links for publicly available datasets
- A description of any restrictions on data availability
- For clinical datasets or third party data, please ensure that the statement adheres to our policy

All data generated for or used within this manuscript have been deposited at Open Science Framework (OSF) and are publicly available here: https://osf.io/ncvpq/?view_only=8fc5fc5aad254fce92a79390ae84b81c

Human research participants

Policy information about studies involving human research participants and Sex and Gender in Research.

Reporting on sex and gender	N/A
Population characteristics	N/A
Recruitment	N/A
Ethics oversight	N/A

Note that full information on the approval of the study protocol must also be provided in the manuscript.

Field-specific reporting

Please select the one below that is the best fit for your research. If you are not sure, read the appropriate sections before making your selection.

☒ Life sciences ☐ Behavioural & social sciences ☐ Ecological, evolutionary & environmental sciences

For a reference copy of the document with all sections, see nature.com/documents/nr-reporting-summary-flat.pdf

Life sciences study design

All studies must disclose on these points even when the disclosure is negative.

Sample size	Sample size was determined based on the variance of early pilot studies and then expanded upon.
Data exclusions	Data was excluded when clear indications of hardware failure had been noted during testing (typically in HD-MEA having faulty electrodes) or later observations during data analysis of spontaneous activity indicated that cultures were probably dead and should not have been subjected to testing.
Replication	The key results in this study have been replicated as reported in the study and cited work over 5 distinct batches of experimental investigations.
Randomization	Not relevant for biological cultures as different cultures received identical treatments as described in the study.
Blinding	Blinding was not possible with this study during data collection as experimenter was required to run specific assays on cells. Blinding was done on data analysis where possible. In most cases this was difficult to implement given the type of analysis performed.

Reporting for specific materials, systems and methods

We require information from authors about some types of materials, experimental systems and methods used in many studies. Here, indicate whether each material, system or method listed is relevant to your study. If you are not sure if a list item applies to your research, read the appropriate section before selecting a response.

Materials & experimental systems

n/a	Involved in the study	
☒	☐	Antibodies
☐	☒	Eukaryotic cell lines
☒	☐	Palaeontology and archaeology
☐	☒	Animals and other organisms
☒	☐	Clinical data
☒	☐	Dual use research of concern

Methods

n/a	Involved in the study	
☒	☐	ChIP-seq
☒	☐	Flow cytometry
☒	☐	MRI-based neuroimaging

Eukaryotic cell lines

Policy information about cell lines and Sex and Gender in Research

Cell line source(s)	Initial work was conducted using a control hIPSC line supplied by the Gene Editing Facility at the Murdoch Children's Research Institute (ATCC PCS-201-010) from an ATCC PCS-201-010 background and transferred under a Material Transfer Agreement. RM3.5 hiPSC line was initially derived from human foreskin fibroblasts and reprogrammed using the hSTEMCCAloxP four

	factor lentiviral vector. Cell lines were generated by Professor Edouard G. Stanley and colleagues from the Murdoch Children's Research Institute and provided under a Material Transfer Agreement.
Authentication	Previous validation of RM3.5 hIPSC line has been performed as cited in text through suing the Illumina HumanCytoSNP-12 v2.1 array and by standard karyotype analysis. ATCC line has been validated as per https://www.atcc.org/products/pcs-201-010
Mycoplasma contamination	All cells were tested and found negative for mycoplasma contamination within 4 weeks of the conclusion of the substantive of the data collection period.
Commonly misidentified lines (See ICLAC register)	No commonly misidentified lines have been used.

Animals and other research organisms

Policy information about studies involving animals; ARRIVE guidelines recommended for reporting animal research, and Sex and Gender in Research

Laboratory animals	BL6/C57 mice were mated and mothers killed when pups were at E15 stage. Cortical cells from all sexes were used as it was not possible to distinguish at this stage.
Wild animals	N/A
Reporting on sex	N/A
Field-collected samples	N/A
Ethics oversight	Animal work was done under ethical approval E/1876/2019/M from the Alfred Research Alliance Animal Ethics Committee B

Note that full information on the approval of the study protocol must also be provided in the manuscript.
